# Prefrontal Blood Flow Activity During Drawing Intervention in School-Age Children with Autism: An fNIRS Hyperscanning Study

**DOI:** 10.3390/brainsci15050438

**Published:** 2025-04-24

**Authors:** Guanghui Li, Daren Wei, Ze Lyu, Yalong Xing, Yan Li, Wu Song

**Affiliations:** 1 Faculty of Innovation and Design, City University of Macau, Macau 999078, China; u22092110172@cityu.edu.mo (G.L.); u23092110264@cityu.edu.mo (D.W.); ruhuayeaimeili@163.com (Z.L.); xingyalong@cityu.mo (Y.X.); 2 College of Mechanical Engineering and Automation, Huaqiao University, Xiamen 361021, China; liyan@hqu.edu.cn

**Keywords:** autism spectrum disorder (ASD), drawing intervention, prefrontal hemodynamics, functional near-infrared spectroscopy (fNIRS)

## Abstract

**Background/Objectives:** Art-based interventions have been shown to enhance communication skills in children with autism spectrum disorder (ASD), yet their impact on prefrontal hemodynamics remains unclear. **Methods:** This study employed functional near-infrared spectroscopy (fNIRS) to examine hemoglobin oxygenation (HbO) changes in the prefrontal cortex of school-age children with ASD, providing empirical support for its therapeutic efficacy. Sixty age-matched children participated in a 9-week art therapy program, including twenty ASD children and forty typically developing peers. Assessments included self-portrait drawing (SPD), the Diagnostic Drawing Series (DDS), and the General Quality of Life Inventory (GQOL-74). In addition, we performed fNIRS measurements in the ASD participants and observed changes in prefrontal HbO at rest and while drawing. **Results:** The drawing intervention significantly enhanced drawing ability, emotional expression, and cognitive skills, with the intervention group outperforming the controls. ASD participants exhibited distinct prefrontal connectivity patterns with visual, motor, and language-related regions, including the dorsolateral prefrontal cortex, frontal eye field, and Broca’s area. Task-based painting interventions indirectly influenced the frontal lobe’s hemodynamic characteristics, indicating drawing intervention as an effective intervention for ASD.

## 1. Introduction

In recent years, the prevalence of autism spectrum disorder (ASD) has been increasing, with a notable 9.5% increase among children aged 3–17. This trend is influenced by various environmental, educational, and sociocultural factors [1]. The core symptoms include audio visual integration defects, which hinder normal social and cognitive development [2,3]. These impairments primarily rely on physical and pharmacological interventions [4], which may cause discomfort due to their highly structured nature. Although existing psychiatric interventions can improve external behaviors, the regulatory mechanisms of intrinsic neurological functions remain a “black box”, and some high-intensity training often triggers resistance in children with ASD. Several studies have indicated that art’s humanistic approach offers an alternative communication and expression intervention that enables individuals to explore their emotions, develop social skills, reduce anxiety, and improve self-esteem. This novel art therapy has proven to be effective in treating this population.

Art therapy offers an alternative means of communication and self-expression, enabling individuals to explore emotions, develop social skills, reduce anxiety, and enhance self-esteem [5]. Among them, vision and hearing are the main channels of artistic intervention. Visual art therapy (such as painting) has been shown to promote social interaction and emotional regulation in people with ASD [6]. Salgado-Vasco has shown that drawing intervention can enhance attention in children with intellectual disabilities, improve language skills in those with specific language impairments, and aid reading accuracy and speed in children with learning disabilities. Kanareff [6] found that group drawing intervention enhances social interactions, improving communication and engagement. In addition, intervention through painting creation has been shown to have a significant regulatory effect on the improvement of patients’ cognition and abilities [7,8,9,10,11].

From an auditory intervention perspective (such as music beats), López-Hernández et al. proposed a music therapy intervention to enhance attention, memory, and language abilities in children with reading disorders, reporting positive outcomes [12]. This type of intervention through auditory art focuses on stimulating and activating brain cells, speeding up the conduction rate in brain nerves, and helping patients relieve emotions and release stress [13]. In summary, art-based interventions are beneficial for people with autism. This personalized and more approachable way of expression helps regulate emotions, reduce pain, enhance emotional expression and improve communication skills [14,15,16,17].

From a neurofunctional perspective, auditory and visual input is essential for the development of advanced skills, such as language, communication, and social interaction [18,19]. By activating the visual-auditory integration of autistic patients, regulating emotions, cultivating motivation, encouragement, comfort, and catharsis, it ultimately alleviates maladaptive behaviors and enhances social adaptability. These effects are consistent with previous findings on the mechanisms of neurofunctional treatment [20].

However, prior studies have primarily focused on behavioral results rather than examining neurophysiological differences in brain function. Despite the rich accumulation of behavioral research (the amount of literature has increased by 120% in the past five years), there are fundamental limitations: 90% of the studies rely only on observation scales, such as the ABC scale, which cannot answer how art therapy changes abnormal brain network connections in ASD. For instance, do different modalities (visual/auditory) activate specific neural pathways? Furthermore, the lack of neural markers makes it impossible to predict the effectiveness of personalized treatment. Moreover, how can the fundamental neural mechanisms of music “auditory stimulation” via music and “visual stimulation” via painting be compared? Which ASD children are more suitable for painting rather than music treatment?

This research gap limits our in-depth understanding of treatment mechanisms and also hinders the development of personalized treatment plans. The research objectives were to explore this neural mechanism, which will provide direct evidence for understanding how art therapy changes abnormal brain network connectivity in ASD and provide neural markers for predicting treatment efficacy. Moreover, we used more ecological fNIRS monitoring technology to synchronize brain function changes in ASD children in painting therapy; detected changes in cerebral oxygenation indirectly related to changes in blood flow, focusing on the hemodynamic response of the prefrontal cortex (executive function), superior temporal gyrus (auditory processing), and occipital cortex (visual processing); and established a corresponding relationship between “behavioral improvement and neural activation”. This study hypothesized that the brain activity of children with autism would change via a drawing process that lasted several weeks, with the activation of specific brain areas in the prefrontal cortex improving their cognitive and behavioral functions.

## 2. Materials and Methods

### 2.1. Participants

Between May and December 2024, a total of 60 age-matched students aged 6–19 years were recruited, comprising 20 children diagnosed with ASD and 40 typically developing (TD) peers. The ASD group’s mean age was 14.86 years (SD = 1.35), with an equal gender distribution (10 males and 10 females). All participants were right-handed. General demographic information was recorded, and informed consent was obtained from the legal guardians of all participants.

The inclusion criteria for ASD participants were as follows: (1) a diagnosis of ASD based on the criteria outlined in the Diagnostic and Statistical Manual of Mental Disorders, Fifth Edition (DSM-5) or the Autism Diagnostic Observation Schedule (ADOS); (2) a diagnosis confirmed by a qualified professional, including a neurologist, psychologist, or psychiatrist; and (3) no first- or second-degree relatives with ASD or related neurodevelopmental disorders (such as ADHD) to reduce genetic confounding [21]. The exclusion criteria for TD children were as follows: (1) the presence of any neurological or developmental disorder or delay, history of preterm birth, or significant perinatal complications; (2) use of neuroactive or psychiatric medications; (3) history of seizures; and (4) family history of ASD.

All participants underwent a 9-week drawing intervention. Pre- and post-intervention assessments were conducted using the Diagnostic Drawing Series (DDS), the Self-Portrait Drawing (SPD) evaluation system, and the General Quality of Life Inventory (GQOL-74).

### 2.2. Drawing Intervention

The drawing intervention consisted of a resting state followed by three distinct task conditions. The experiment was conducted in a quiet and comfortable environment to minimize external disturbances. To ensure the children’s compliance with the protocol, they were instructed to sit on a stool, refrain from speaking or making unnecessary movements, and remain as still as possible throughout the test. The “House Tree Person” (HTP) drawing projection was developed from Buck’s “Draw a Tree Test”, in which subjects are asked to draw a house, a tree, and a person on three blank sheets of paper to complete the test. The dynamic house, tree, and person analysis proposed by Robert Burn in 1970 requires that houses, trees, and people be drawn on the same sheet of paper [22]. These three interact with each other. For example, the location and distance of houses and people can provide information about the subject’s relationship to the home, so the analysis of both can be used in conjunction.

**Figure 1:**
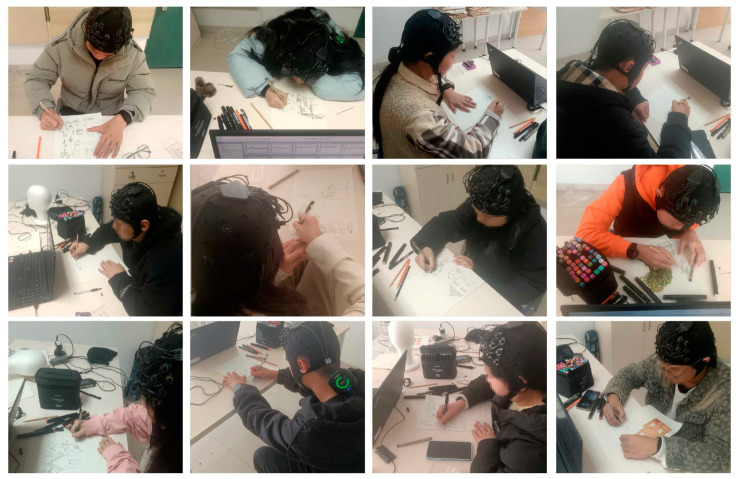
Examples of drawing intervention test situations in 3 task states.

**Figure A1:**
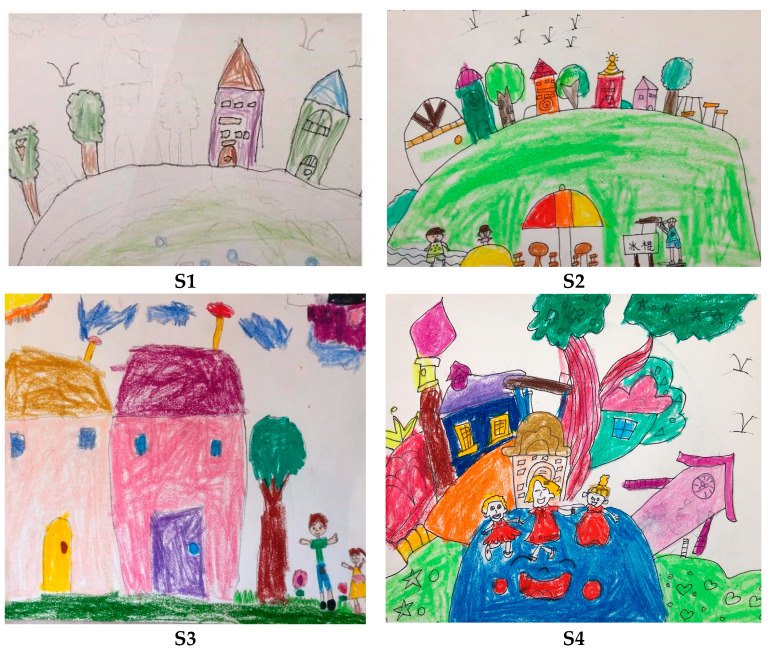
The HTP task-based intervention process, the transformation of one author’s drawing style within a few weeks. S1: The author struggled with ideas and confidence when experimenting with painting materials. There are large blank areas and repeatedly rubbed marks. Being introverted and having difficulty expressing himself, it was also difficult to establish good communication. S2: After several days of practice, the author gradually gained confidence, which was reflected in his works with themes such as trees, houses, seagulls, grasslands, etc. Notably, the image also features the Chinese word “冰棍” (bīng gùn, meaning “ice pop” or “popsicle”), the characters in the scene show him standing contentedly. S3: The author completed this painting silently according to the natural landscape and emphasized his own feelings. The author emphasizes expressing one’s feelings and learning to express oneself through painting language. S4: A few weeks later, the author felt his body gradually recover. The author is more confident in drawing himself in the center, using more colors to describe the harmonious scenes of spring that he saw, making new friends, and describing the warmth and happiness he felt with a big smile.

A 120 s interval was maintained between each painting session to prevent potential carryover effects between tasks. During the sessions, the participants sat on a stool positioned 50 cm away from the table and engaged in the painting activities (Figure 1). Both groups of participants drew a house, a tree, and a person in sequence. The painting intervention was a 9-week longitudinal experiment that lasted for 9 weeks, with one treatment per week. The behavioral results were compared in the initial collection in the first week and the final collection in the ninth week. fNIRS data were collected for each ASD patient in turn. We show the change in the drawing style of one of the authors and the effect of the intervention in Appendix A (Figure A1).

### 2.3. fNIRS Testing

**Figure 2:**
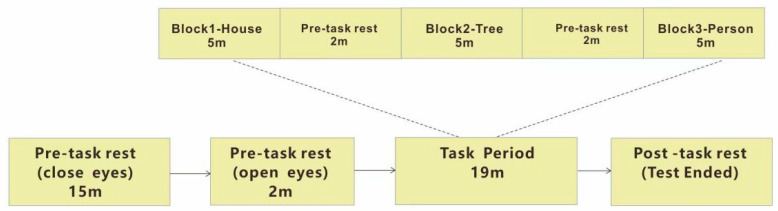
VFT task sequence in drawing intervention.

fNIRS assessments were conducted under two conditions: resting state and drawing intervention sessions. The resting-state measurement lasted for 15 min, including a 2 min break. The drawing intervention followed a block design, with each session lasting 19 min and incorporating a 2 min rest period between visual painting tasks. Both groups underwent fNIRS resting-state testing and participated in three distinct drawing intervention tasks: drawing a house, a tree, and a person. Over six weeks, each child completed six fNIRS assessments. A 2 min interval was maintained between consecutive visual painting sessions to minimize potential cross-task interference. Additionally, the order of the three tasks was randomized. The experimental process is shown in Figure 2.

The fNIRS measurements were conducted using the Brite23 device (Artinis Medical Systems, Elst, Gelderland, The Netherlands). The device operated with a sampling rate of 10 Hz and was configured with 24 measurement channels. Dual-wavelength detection was employed at 760 nm and 850 nm, with a fixed source-detector distance of 3 cm. Brain activity was assessed by converting absorbance variations into the relative concentration changes in oxyhemoglobin (HbO). Previous research has consistently demonstrated that HbO exhibits a higher signal-to-noise ratio (SNR) compared with deoxygenated hemoglobin (HbR), making it more suitable for detecting task-related hemodynamic changes, especially in pediatric populations [23]. While HbR is known to provide better spatial specificity and may reflect localized neuronal deactivation, its lower amplitude and higher susceptibility to systemic noise limit its reliability in short-duration tasks or studies involving younger participants. Given our study’s design and signal quality, we prioritized HbO to ensure consistent and interpretable measurements of cortical activation, which shows the strongest positive correlation with the BOLD signal in fMRI [24].

**Figure 3:**
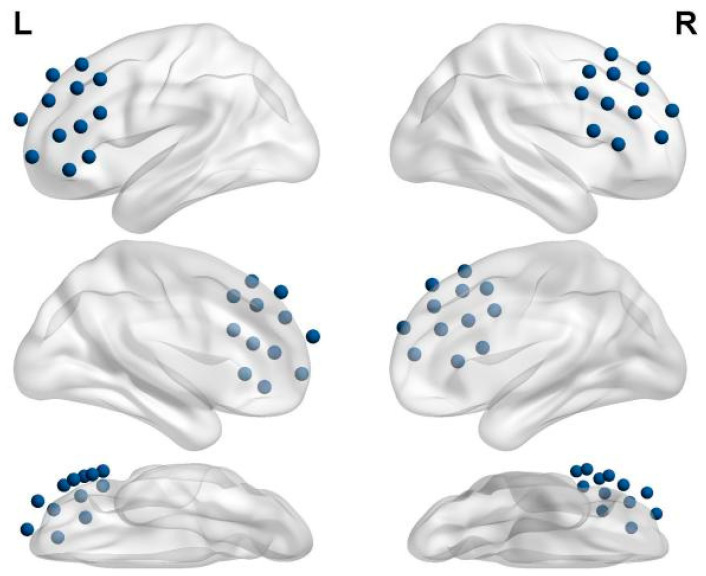
Topographical layout of fNIRS optodes over the bilateral prefrontal cortex. Left one, left-side plan view of the left-brain template; left two, right-side plan view of the left-brain template; left three, top view of the left-brain template; right one, right-side plan view of the right-brain template; right two, left-side plan view of the right-brain template; right three, top view of the right-brain template.

The fNIRS optodes were positioned following the international 10–20 system to ensure accurate coverage of the prefrontal cortex, as illustrated in Figure 3. A total of 18 optodes (10 emitters and 8 detectors) were arranged in a 2 × 12 bilateral grid configuration, yielding 24 measurement channels based on emitter–detector pairings with a 3 cm inter-optode distance. The probabilistic mapping of these 24 channels onto Brodmann areas was calculated using a 3D localization system. This arrangement allowed for targeted recording in key regions of interest (ROIs), including the dorsolateral prefrontal cortex (DLPFC), frontopolar cortex (FPC), and orbitofrontal cortex (OFC), which are implicated in emotional regulation, executive function, and social cognition.

### 2.4. Statistical Analysis

#### 2.4.1. Demographic and Clinical Data

The effectiveness of the drawing intervention was assessed using the SPD and DDS evaluation systems [25,26,27] and the GQOL-74 scale [23]. Data analysis and visualization were performed using IBM SPSS Statistics for Windows, Version 27.0 (IBM Corp., Armonk, NY, USA) and Python 3.9. Pearson correlation was used to examine the associations between color usage, line quality, and spatial occupancy, while a paired *t*-test assessed the pre- and post-intervention differences in artistic expression and psychological development in children with ASD.

#### 2.4.2. fNIRS Processing

A Polhemus 3D digitizer measured and standardized the fNIRS channel coordinates for 20 participants. The correspondence between channels and brain regions was determined using the NIRS-SPM data analysis package of MATLAB 2014. Channel-to-brain region mapping was determined via probabilistic calculations in SPM. Functional connectivity (FC) matrices were computed using SPM, followed by a paired t-test to compare the resting state and drawing intervention conditions. The data-processing steps included (1) converting oxy4 format data; (2) detrending to remove data trends or linear drift (optical density values converted to blood oxygen values); (3) applying an IRR filter for signal processing (0.001–0.08Hz); (4) computing FC matrices for both the resting state and task state at the individual level (Fisher z-score using Pearson correlation); and (5) conducting paired t-tests to examine the functional connectivity differences between the resting state and drawing intervention sessions. The purpose was to reveal the regulatory mechanism of the task on functional connectivity and explore the dynamic changes in task-related neural networks (for example, the drawing task enhanced the functional connectivity related to the task (such as the prefrontal–parietal network) while inhibiting the functional connectivity of non-task-related networks (such as the default mode network)).

## 3. Results

### 3.1. Drawing Intervention Enhances Self-Perception in ASD

In this study, self-portrait drawings were used to assess self-awareness and emotional expression. The DDS, developed by Cohen in 1983, consists of three drawing tasks performed over 90 min. Researchers evaluated 68 indicators in the artwork to compute the DDS scores. Higher scores indicate a stronger likelihood of autism. The DDS assesses both painting performance and psychological characteristics, shedding light on cognitive processes, emotional expression, and interactions with the environment in autistic individuals.

**Figure 4:**
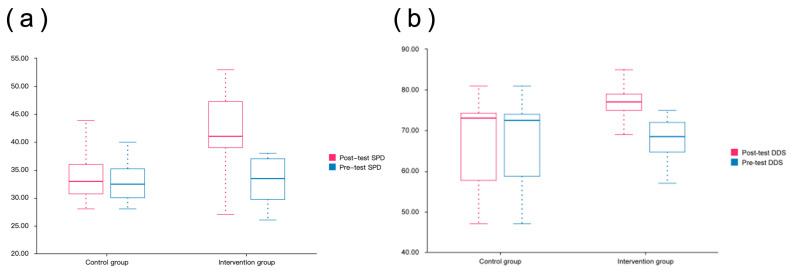
(**a**) Box plot of SPD scores and (**b**) box plot of DDS scores.

As shown in Figure 4, the scores of the intervention group after the intervention were significantly higher than those before the intervention, and the intervention group showed a significant improvement in the post-test compared with the control group. The self-perception of the intervention group (SPD assessed by self-portrait drawing) showed a greater improvement after the intervention, while the control group had a smaller change between the pre- and post-tests and no significant difference.

The score distributions of the control group and the intervention group showed a significant difference. The DDS score of the intervention group in the post-test was higher than that in the pre-test, and the change in the score was larger, showing the positive effect of the intervention on the students’ painting performance. In contrast, the scores of the control group changed less between the pre- and post-tests, and the overall scores remained at a low level. This shows that the intervention may have significantly improved the painting performance of students with autism, especially in cognition, emotional expression, and interactions with the environment.

#### 3.1.1. SPD Analysis

As shown in Table 1, the pre-test SPD scores showed no significant difference between the control and intervention groups (*t* = 0.109; *p* = 0.914). However, the post-test SPD scores were significantly higher in the intervention group compared with the control group (*t* = −4.763; *p* < 0.01), indicating that the painting intervention had a positive impact on self-cognition and emotional expression in children with ASD, particularly within the intervention group. The post-test SPD score of the intervention group (41.77 ± 6.85) was substantially higher than the pre-test score (32.93 ± 3.69), reflecting an increase of 8.83 points. A paired-sample *t*-test revealed a significant improvement in self-cognition through painting (*t* = 7.387; *p* < 0.001). These results confirm that painting intervention is effective in enhancing self-cognition in children with ASD, promoting their emotional, cognitive, and self-expressive development.

#### 3.1.2. DDS Analysis

There was no significant difference between the control and intervention groups in the pre-test DDS scores (*t* = 0.433; *p* = 0.667). However, the post-test results show that the DDS score in the intervention group was significantly higher than that in the control group (*t* = −3.041; *p* < 0.01), indicating that the intervention effectively enhanced the drawing performance of children with ASD. The post-test DDS score in the intervention group (75.30 ± 6.88) was notably higher than the pre-test score (67.67 ± 5.02), showing an increase of 7.63 points. A paired-sample *t*-test yielded a t-value of 10.419 and a *p*-value of 0.000 (*p* < 0.01), confirming significant improvements in artistic expression and psychological well-being. These findings suggest that art intervention positively influenced both painting skills and psychological development in children with ASD, enhancing multiple abilities and demonstrating superior effectiveness compared with the control group. Additionally, the DDS results emphasize the role of color and spatial elements in artistic expression, aligning with Bernier’s conclusions [28].

#### 3.1.3. GQOL-74 Analysis

The GQOL-74 questionnaire is widely used to assess quality of life in both the general population and specific groups. It consists of 74 questions covering 20 factors across four dimensions: physical function, psychological function, social function, and material living conditions. Since this study focused on children with ASD, whose material living conditions are generally stable, the material living status dimension was excluded. The modified GQOL-74 scale used in this study included 14 factors across three dimensions: physical function, psychological function, and social function. Additionally, given that participants were aged 6–19 years, the sexual function factor within the physical function dimension was omitted, resulting in a total of 64 questions.

**Figure 5:**
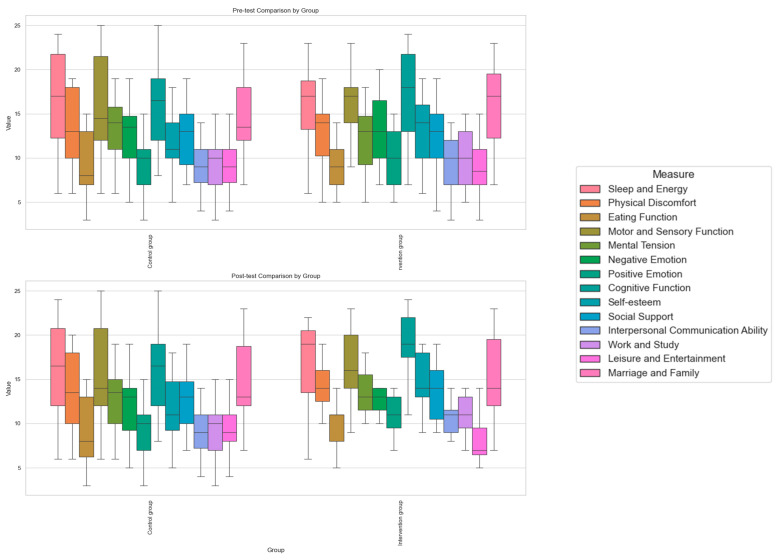
The box plots of the pre-test (**top**) and post-test (**bottom**) of the GQOL-74 scale.

Figure 5 presents the pre-test results of the GQOL-74 questionnaire, showing differences between the control and intervention groups across various dimensions. While both groups exhibited similar distributions in physical, psychological, and social functions with no significant differences, the intervention group demonstrated a more concentrated distribution in certain aspects, particularly in negative emotions and cognitive functions. This highlights individual variability among autistic students. Overall, the pre-test results indicate minimal differences in overall quality of life between the groups but reveal considerable individual variation across dimensions, with notable fluctuations in emotion and cognition within the intervention group.

The post-test results show significant improvements in multiple dimensions within the intervention group, particularly in negative emotions, positive emotions, and cognitive functions. Compared with the pre-test, the increased spread of the box plots in the intervention group suggests a positive impact of drawing intervention on emotional regulation, cognitive abilities, and social functioning in autistic students. In contrast, the control group exhibited minimal changes, with no substantial improvements observed in most dimensions of quality of life. These findings suggest that painting intervention effectively enhances the overall quality of life of autistic students, with particularly pronounced benefits in emotional and cognitive functions.

In Table 2, the intervention group outperformed the control group across all indicators, with significant differences observed in “overall life satisfaction” and “health status”. The intervention group scored 3.47 for overall life satisfaction and 3.7 for health status, compared with 3.0 and 3.1 in the control group, respectively, indicating a clear improvement. However, for “overall satisfaction with own health”, the difference between the intervention (3.57) and control (3.13) groups was not statistically significant. Overall, the painting-based intervention significantly enhanced the life satisfaction and health status of children with ASD, though its impact on self-perceived health satisfaction did not reach statistical significance.

The intervention group showed higher scores for multiple indicators compared with the control group, with some differences being statistically significant. While the intervention did not significantly improve “sleep and energy levels”, “eating function”, or “motor and sensory function”, it had a notable effect on alleviating “physical discomfort” (15.03 in the intervention group vs. 13.1 in the control group). In summary, the painting-based intervention effectively reduced physical discomfort but had a limited impact on other physical functions.

In terms of psychological function, the intervention group scored higher than the control group. Although the intervention showed no significant effect on “mental stress” (*t* = −1.545; *p* = 0.128), it significantly improved “negative emotions”, “positive emotions”, and “cognitive function” (*t* = −2.552, −2.252, and −2.902; *p* = 0.013, 0.028, and 0.005, respectively). Overall, the painting-based intervention effectively reduced negative emotions, enhanced positive emotions, and improved cognitive function, but had limited effectiveness in relieving mental stress.

Following the *t*-test, there was no significant difference between the control group and the intervention group before the intervention (*p* > 0.05), indicating that the two groups started from similar baseline levels. After the painting intervention, the comprehensive quality of life of ASD children improved in various dimensions:

- Emotional psychology: Negative and positive emotions, self-esteem, and cognitive functions were significantly improved (*p*-values all < 0.05), indicating enhanced emotional regulation, self-confidence, and cognitive abilities.
- Social function: Interpersonal communication and work/study abilities were significantly improved (*p* < 0.05), and social support showed a positive trend but did not reach significance (*p* = 0.083).
- Physical function: Physical discomfort was significantly improved (*p* = 0.04), but no significant improvements were observed in sleep, eating, or motor–sensory functions (*p* > 0.05).
- Other dimensions: Marital and family relationships were significantly improved (*p* = 0.027), while no significant changes were found in leisure and entertainment (*p* = 0.254).

In summary, painting intervention has a positive effect on the quality of life of ASD children, especially in terms of emotion, cognition, and social interaction. Although some physical functions were not significantly improved, the intervention still promoted their social and self-identity development.

After the intervention, ASD children significantly improved in multiple dimensions, especially in emotional psychology and social skills. In the emotional psychology dimension, indicators such as sleep energy, physical discomfort, mental tension, positive and negative emotions, cognitive function, and self-esteem were significantly improved (*p* < 0.05), indicating that the intervention improved energy, regulated emotions, and enhanced cognition and self-confidence. In terms of social function, social support, interpersonal communication, work and study, and amateur entertainment were significantly improved (*p* < 0.05), indicating that the intervention enhanced social skills and improved multi-faceted participation. There was an improvement trend in marriage and family (*p* = 0.052), but it did not reach a significant level. In conclusion, art intervention has a positive impact on the quality of life of children with ASD, and drawing intervention such as painting is conducive to their emotional expression and social interaction, which has been confirmed by relevant studies [28,29].

### 3.2. Drawing Intervention Facilitates Visual and Expressive Functions in ASD

Using traditional drawing intervention assessment criteria combined with survey analysis, we found that the subjects exhibited significant improvements in all aspects after 9 weeks of drawing intervention. Drawing intervention undoubtedly played a positive role. Among them, the prefrontal cortex, the most evolutionarily recent and functionally critical brain structure in higher cognitive processes, exhibited physiological changes during the drawing intervention that held direct and significant explanatory value. A single channel is formed between a light-emitting optode and a detector, representing the observed changes in the prefrontal cortex. Utilizing a 2 × 12 optode template, which includes 10 emitters and 8 detectors, a total of 24 channels were established. The probabilistic mapping of these 24 channels onto Brodmann areas was calculated using a 3D localization system, as shown in Table 3.

The functional differences between the resting state and the drawing intervention process were significantly reflected in the following three channels: (1) functional connectivity differences between participants in prefrontal associational integration and the dorsolateral prefrontal cortex and primary visual cortex responsible for visual information processing; (2) participants in the prefrontal associational integration frontopolar area (the most rostral part of the superior and middle frontal gyri) and “eye movement control (including the frontal eye field)”; and (3) the frontopolar area (the most rostral part of the superior and middle frontal gyri) of the dorsolateral prefrontal cortex and the “pars triangularis, part of Broca’s area”.

**Figure 6:**
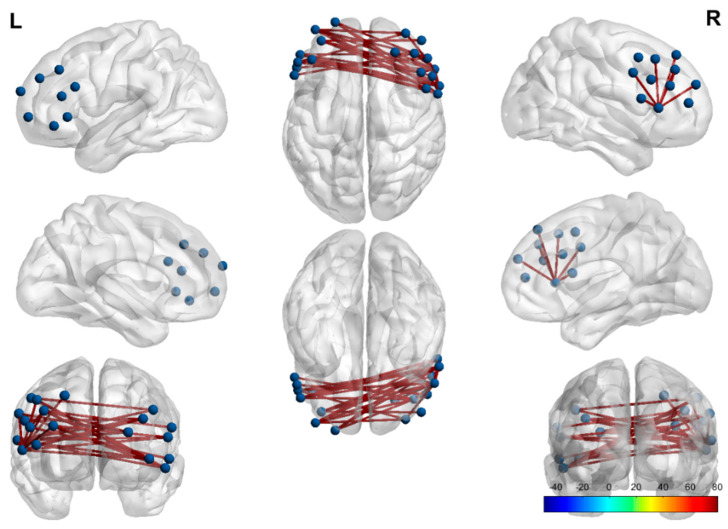
Differences in functional connectivity between the 24 channels and the corresponding brain regions in the resting state and during the drawing intervention.

This shows that during the drawing intervention, the prefrontal lobe and the inferior temporal gyrus for visual information processing, the Broca area for language production, and the eye movement control area of the autistic group all significantly “moved”. Previous studies have found differences in audiovisual and speech integration between autistic and non-autistic individuals [20]. In addition, studies have shown how multisensory integration defects may also be the core characteristics of ASD [30,31], and multisensory integration ability is an evaluation indicator in autism assessment. More broadly, the observed activation patterns suggest that the drawing intervention may enhance the coordination of multisensory integration processes—such as the coupling of visual–motor and affective–cognitive functions—thereby contributing to improved neural efficiency and potential therapeutic effects for individuals with ASD (Figure 6).

Differences in functional connectivity between the inferior temporal gyrus, the eye movement control area, and the prefrontal lobe reflect the integration of visual and executive functions. Similarly, differences in connectivity between Broca’s area and the prefrontal lobe indicate variations in speech production and language processing. From this point of view, it seems more reasonable to explain the effect of drawing intervention as promoting audio-visual speech integration. Individuals exhibit substantial variability in their propensity for audiovisual integration [32]. This difference is particularly evident when comparing the general population with certain clinical populations, including those with autism [33,34]. Some “sensory first” theories propose that sensory differences play a crucial role in neurodevelopmental conditions such as ASD, primarily due to variations in perception [35,36,37,38]. The hemodynamic responses observed during drawing intervention align with previous behavioral findings [39]. Most individuals with ASD exhibit delays in language acquisition and persistent atypical responses to speech stimuli. Research has concluded that individuals with ASD do not improve in audiovisual speech integration, which in turn widens the gap between them and neurotypical individuals, who continue to develop this ability into adulthood [40]. In 2015, Foxe and Taylor suggested that differences in audiovisual speech integration between ASD and neurotypical children may be resolved during adolescence [3,41]. Irrespective of the prevailing perspective, these viewpoints collectively suggest that audiovisual speech integration is crucial in ASD, underscoring its therapeutic relevance.

## 4. Discussion

fNIRS exhibits a high spatial resolution, enabling finer differentiation of brain regions compared with electroencephalography (EEG). The results’ analysis found that there were also some special functional connectivity differences between the prefrontal brain regions. Biological evidence suggests that analyzing synaptic components in the prefrontal cortex could reveal proteomic features serving as biomarkers for the biological basis of cognitive deficits in children with ASD [42]. Our observations present the following within the same prefrontal cortex: (1) differences in functional connectivity between the frontopolar area (the most rostral part of the superior and middle frontal gyri) and the dorsolateral prefrontal cortex (DLPFC); (2) differences in functional connectivity between the prefrontal associational integration (the inferior frontal gyrus) and the dorsolateral prefrontal cortex. Specifically, (1) activating the DLPFC improves patients’ ability to regulate emotions, thereby enhancing their ability to manage their own emotions. (2) The process of artistic creation requires planning, execution, and memory (such as the imitation and association of real scenes and the re-expression of color), which directly exercises the working memory and executive functions dominated by the DLPFC. (3) Self-reflection and social interaction abilities are significantly enhanced. Paintings can directly reflect patients’ shaping of self-awareness (self-belief) and partner image shaping (team awareness), and patients’ empathy is significantly improved, which is due to the enhanced social interaction regulation of the DLPFC. We found that the frontopolar region and the inferior frontal gyrus exhibited significant differences in functional connectivity with the dorsolateral prefrontal cortex during the drawing intervention. Furthermore, some researchers consider the characteristics of the dorsolateral prefrontal cortex PFC to be the key to distinguishing ASD from ADHD [43,44]. These findings collectively suggest that the dorsolateral prefrontal cortex (DLPFC) plays a distinct role in the neural function of individuals with autism. Previous research has extensively investigated the efficacy of transcranial magnetic stimulation (TMS) targeting the left DLPFC in high-functioning ASD individuals. This also confirms the fMRI research results. The process of artistic creation requires planning, execution, and memory (such as the imitation and association of real scenes and the re-expression of color), which directly exercises the working memory and executive functions dominated by the DLPFC [45]. Cognitive behavioral therapy (CBT) strengthens DLPFC amygdala connectivity, which mediates emotional control [46]. Casanova, Sokhadze, and colleagues administered repetitive TMS to the DLPFC in individuals with ASD, delivering stimulation twice weekly. After three weeks, their findings demonstrated statistically significant reductions in repetitive behaviors [47,48]. Furthermore, a separate review reported that cortical theta-burst stimulation (CTB) of the left DLPFC may enhance planning and decision-making processes in both neurotypical adults and individuals with treatment-resistant depression [49]. The DLPFC plays a pivotal role in various higher-order cognitive functions. While there has been ongoing debate regarding the efficacy of stochastic stimulation of the DLPFC in activating and improving ASD-related symptoms, our findings suggest that painting intervention facilitated functional connectivity within the prefrontal cortex, with the DLPFC exhibiting a dominant role in these neural interactions. Art therapy’s efficacy in alleviating ASD symptoms may stem from its role in enhancing the regulatory capacity of the DLPFC, thereby improving cognitive and behavioral regulation within the prefrontal network.

## 5. Conclusions

The efficacy of drawing intervention in school-age children with ASD was assessed over a 9-week intervention period using standardized art therapy assessment criteria and questionnaire-based assessments. Additionally, fNIRS was employed to monitor prefrontal cerebral blood flow changes, revealing significant improvements in neural function. Integrating these findings with the prior literature, painting intervention can effectively enhance the visual and auditory language integration ability of school-age children with autism by exercising the dorsolateral prefrontal cortex’s regulatory ability on the inferior frontal gyrus and prefrontal area, which is consistent with the results of previous studies. The study provides a more scientific explanation for future personalized and creative painting interventions to improve the performance of ASD school-age children.

## Figures and Tables

**Table 1:** *t*-test results for pre- and post-test (**a**) SPD and (**b**) DDS scores in control and intervention groups. ** indicates *p* < 0.01.

**Independent-Samples *t*-Test**					**Paired-Samples *t*-Test**				
**(a) SPD**	**Group (Mean ± SD)**		***t***	***p***	**Paired (Mean ± SD)**		**Difference**	***t***	***p***
	Control group (n = 30)	Intervention group (n = 30)							
Pre-test	33.03 ± 3.41	32.93 ± 3.69	0.109	0.914	Post-test	Pre-test			
Post-test	34.13 ± 5.49	41.77 ± 6.85	−4.763	0.000 **	41.77 ± 6.85	32.93 ± 3.69	8.83	7.387	0.000 **
**(b** **) DDS**	**Group (mean ± SD)**		***t***	***p***	**Paired (mean ± SD)**		**Difference**	***t***	***p***
	Control group (n = 30)	Intervention group (n = 30)							
Pre-test	68.50 ± 9.28	67.67 ± 5.02	0.433	0.667	Post-test	Pre-test			
Post-test	68.67 ± 9.76	75.30 ± 6.88	−3.041	0.004 **	75.30 ± 6.88	67.67 ± 5.02	7.63	10.419	0.000 **

**Table 2:** Overall post-test comparison between the intervention and control groups.

Features	Group	Sample Size	Mean	SD	Standard Error of Mean	t	Significance (Two-Tailed)
Health satisfaction	Control	30	3.13	1.042	0.19	−1.758	0.084
	Intervention	30	3.57	0.858	0.157		
Life satisfaction	Control	30	3	0.695	0.127	−2.454	0.017
	Intervention	30	3.47	0.776	0.142		
Health status	Control	30	3.1	0.759	0.139	−2.909	0.005
	Intervention	30	3.7	0.837	0.153		
Sleep and energy	Control	30	15.93	5.356	0.978	−1.294	0.201
	Intervention	30	17.5	3.911	0.714		
Physical discomfort	Control	30	13.1	4.326	0.79	−2.108	0.039
	Intervention	30	15.03	2.553	0.466		
Eating function	Control	30	9.3	3.659	0.668	−0.895	0.375
	Intervention	30	10	2.228	0.407		
Motor and sensory functions	Control	30	15.63	5.493	1.003	−1.584	0.12
	Intervention	30	17.53	3.608	0.659		
Mental stress	Control	30	12.77	3.803	0.694	−1.545	0.128
	Intervention	30	14.1	2.808	0.513		
Negative emotions	Control	30	11.97	3.846	0.702	−2.552	0.013
	Intervention	30	14.23	2.979	0.544		
Positive emotion	Control	30	9.17	3.26	0.595	−2.252	0.028
	Intervention	30	10.87	2.543	0.464		
Cognitive function	Control	30	16	4.749	0.867	−2.902	0.005
	Intervention	30	19.1	3.418	0.624		

**Table 3:** Brodmann partitions and probabilities of 24 channels.

No.	Anatomical Label	Percentage of Overlap
CH01: 47	Inferior prefrontal gyrus	0.97627
CH02: 10	Frontopolar area	0.97308
CH03: 46	Dorsolateral prefrontal cortex	0.74909
CH04: 47	Inferior prefrontal gyrus	0.67742
CH05: 45	Dorsolateral prefrontal cortex	0.66782
CH06: 45	Pars triangularis Broca’s area	0.45424
CH07: 10	Frontopolar area	1
CH08: 10	Frontopolar area	0.46637
CH09: 9	Dorsolateral prefrontal cortex	0.62996
CH10: 8	Includes frontal eye fields	0.50193
CH11: 8	Includes frontal eye fields	0.6009
CH12: 8	Includes frontal eye fields	1
CH13: 45	Pars triangularis Broca’s area	0.64762
CH14: 10	Frontopolar area	0.55056
CH15: 46	Dorsolateral prefrontal cortex	0.98901
CH16: 44	Pars opercularis, part of Broca’s area	0.59091
CH17: 9	Dorsolateral prefrontal cortex	0.57143
CH18: 6	Pre-motor and supplementary motor cortex	0.51449
CH19: 10	Frontopolar area	0.89831
CH20: 9	Dorsolateral prefrontal cortex	0.72093
CH21: 8	Includes frontal eye fields	0.67886
CH22: 6	Pre-motor and supplementary motor cortex	0.43952
CH23: 8	Includes frontal eye fields	0.91915
CH24: 8	Includes frontal eye fields	0.80242

## Data Availability

The original contributions presented in the study are included in the article. Further inquiries can be directed to the corresponding author.

## References

[1] Zablotsky B., Black L.I., Maenner M.J., Schieve L.A., Danielson M.L., Bitsko R.H., Blumberg S.J., Kogan M.D., Boyle C.A. (2019). Prevalence and Trends of Developmental Disabilities among Children in the United States: 2009–2017. Pediatrics.

[2] Feldman J.I., Dunham K., DiCarlo G.E., Cassidy M., Liu Y., Suzman E., Williams Z.J., Pulliam G., Kaiser S., Wallace M.T. (2023). A Randomized Controlled Trial for Audiovisual Multisensory Perception in Autistic Youth. J. Autism Dev. Disord..

[3] Taylor N., Isaac C., Milne E. (2010). A comparison of the development of audiovisual integration in children with autism spectrum disorders and typically developing children. J. Autism Dev. Disord..

[4] Sharma S.R., Gonda X., Tarazi F.I. (2018). Autism Spectrum Disorder: Classification, diagnosis and therapy. Pharmacol. Ther..

[5] Livengood de Sanabria M.d.l.Á. (2022). Musicoterapia en infantes: Funciones cognitivas y emociones. Rev. Cuba. Pediatría.

[6] Kanareff R.L. (2002). Utilizing Group Art Therapy to Enhance the Social Skills of Children with Autism and Down Syndrome.

[7] Zhang W. (2009). Multi-Factor Investigation and Analysis of Autistic Children and Intervention of Art Therapy. Master’s Thesis.

[8] Cui J., Xie X. (2013). Experimental study on art therapy intervention for children with autism. J. Tangshan Norm. Univ..

[9] Liang Y. (2016). Analysis of multi-factor investigation and intervention of art therapy for autistic children. Art Technol..

[10] Shen W. (2017). A Case Study of Art Education Therapy for Emotionally Disturbed Children. Master’s Thesis.

[11] Yu S., Lin L. (2017). Experimental study on the intervention therapy of drawing for children with autism. Art Technol..

[12] López-Hernández E., Acosta-Rodas P., Cruz-Cárdenas J., Ramos-Galarza C. (2021). Intervención musicoterapéutica para mejorar la memoria, atención y lenguaje in niños con dislalia. Rev. Ecuat. Neurol..

[13] Chaieb L., Wilpert E.C., Reber T.P., Fell J. (2015). Auditory beat stimulation and its effects on cognition and mood States. Front. Psychiatry.

[14] Han Y.M.Y., Chan M.C., Chan M.M.Y., Yeung M.K., Chan A.S. (2022). Effects of working memory load on frontal connectivity in children with autism spectrum disorder: A fNIRS study. Sci. Rep..

[15] D'Amico M., Lalonde C. (2017). The Effectiveness of Art Therapy for Teaching Social Skills to Children with Autism Spectrum Disorder. Art Ther..

[16] Kim T.H., Li E.O.I. (2018). Mandala art therapy: Intervention for individual with autism spectrum disorder (ASD). J. Psikol. Malays..

[17] Harris C. (2015). Portrait Drawing: An Art Therapy Intervention for Adults with Autism Spectrum Disorder. Master’s Thesis.

[18] MacDonald K., Marchman V.A., Fernald A., Frank M.C. (2020). Children flexibly seek visual information to support signed and spoken language comprehension. J. Exp. Psychol. Gen..

[19] Rubio-Fernandez P., Mollica F., Jara-Ettinger J. (2021). Speakers and listeners exploit word order for communicative efficiency: A cross-linguistic investigation. J. Exp. Psychol. Gen..

[20] Jertberg R.M., Wienicke F.J., Andruszkiewicz K., Begeer S., Chakrabarti B., Geurts H.M., de Vries R., Van der Burg E. (2024). Differences between autistic and non-autistic individuals in audiovisual speech integration: A systematic review and meta-analysis. Neurosci. Biobehav. Rev..

[21] Rommelse N.N., Franke B., Geurts H.M., Hartman C.A., Buitelaar J.K. (2010). Shared heritability of attention-deficit/hyperactivity disorder and autism spectrum disorder. Eur. Child Adolesc. Psychiatry.

[22] Burns R.C., Kaufman S.H. (2013). Action, Styles, and Symbols in Kinetic Family Drawings KFD.

[23] Ferrari M., Quaresima V. (2012). A brief review on the history of human functional near-infrared spectroscopy (fNIRS) development and fields of application. NeuroImage.

[24] Broder-Fingert S., Feinberg E., Silverstein M. (2017). Music therapy for children with autism spectrum disorder. JAMA.

[25] Martin N. (2008). Assessing portrait drawings created by children and adolescents with autism spectrum disorder. Art Ther..

[26] Shen K.-S., Chen K.-H., Liang C.-C., Pu W.-P., Ma M.-Y. (2012). Measuring the functional and usable appeal of crossover B-Car interiors. Hum. Factors Ergon. Manuf. Serv. Ind..

[27] Kring A.M., Smith D.A., Neale J.M. (1994). Individual differences in dispositional expressiveness: Development and validation of the Emotional Expressivity Scale. J. Pers. Soc. Psychol..

[28] Bernier A., Ratcliff K., Hilton C., Fingerhut P., Li C.Y. (2022). Art Interventions for Children With Autism Spectrum Disorder: A Scoping Review. Am. J. Occup. Ther..

[29] Baek D., Baek J., Noh J., Oh Y., Lim L. (2024). Toward Healthy Underground Spaces: A Review of Underground Environmental Design Factors and Their Impacts on Users’ Physiological and Psychological Health. HERD Health Environ. Res. Des. J..

[30] Baum S.H., Stevenson R.A., Wallace M.T. (2015). Behavioral, perceptual, and neural alterations in sensory and multisensory function in autism spectrum disorder. Prog. Neurobiol..

[31] Noel J.P., Lytle M., Cascio C., Wallace M.T. (2018). Disrupted integration of exteroceptive and interoceptive signaling in autism spectrum disorder. Autism Res..

[32] Magnotti J.F., Beauchamp M.S. (2018). Published estimates of group differences in multisensory integration are inflated. PLoS ONE.

[33] Stevenson R.A., Siemann J.K., Schneider B.C., Eberly H.E., Woynaroski T.G., Camarata S.M., Wallace M.T. (2014). Multisensory temporal integration in autism spectrum disorders. J. Neurosci..

[34] Brandwein A.B., Foxe J.J., Butler J.S., Russo N.N., Altschuler T.S., Gomes H., Molholm S. (2013). The development of multisensory integration in high-functioning autism: High-density electrical mapping and psychophysical measures reveal impairments in the processing of audiovisual inputs. Cereb. Cortex.

[35] Cascio C.J., Woynaroski T., Baranek G.T., Wallace M.T. (2016). Toward an interdisciplinary approach to understanding sensory function in autism spectrum disorder. Autism Res..

[36] Uljarevic M., Baranek G., Vivanti G., Hedley D., Hudry K., Lane A. (2017). Heterogeneity of sensory features in autism spectrum disorder: Challenges and perspectives for future research. Autism Res..

[37] Green D., Chandler S., Charman T., Simonoff E., Baird G. (2016). Brief Report: DSM-5 Sensory Behaviours in Children With and Without an Autism Spectrum Disorder. J. Autism Dev. Disord..

[38] Feng S., Wang Q., Hu Y., Lu H., Li T., Song C., Fang J., Chen L., Yi L. (2023). Increasing audiovisual speech integration in autism through enhanced attention to mouth. Dev. Sci..

[39] Brignell A., Morgan A.T., Woolfenden S., Klopper F., May T., Sarkozy V., Williams K. (2018). A systematic review and meta-analysis of the prognosis of language outcomes for individuals with autism spectrum disorder. Autism Dev. Lang. Impair..

[40] Zhang J., Meng Y., He J., Xiang Y., Wu C., Wang S., Yuan Z. (2019). McGurk Effect by Individuals with Autism Spectrum Disorder and Typically Developing Controls: A Systematic Review and Meta-analysis. J. Autism Dev. Disord..

[41] Foxe J.J., Molholm S., Del Bene V.A., Frey H.P., Russo N.N., Blanco D., Saint-Amour D., Ross L.A. (2015). Severe multisensory speech integration deficits in high-functioning school-aged children with Autism Spectrum Disorder (ASD) and their resolution during early adolescence. Cereb. Cortex.

[42] Fatemi S.H., Eschenlauer A., Aman J., Folsom T.D., Chekouo T. (2024). Quantitative proteomics of dorsolateral prefrontal cortex reveals an early pattern of synaptic dysmaturation in children with idiopathic autism. Cereb. Cortex.

[43] Li Y., Ma S., Zhang X., Gao L. (2024). ASD and ADHD: Divergent activating patterns of prefrontal cortex in executive function tasks?. J. Psychiatr. Res..

[44] Wang Z., Jing J., Igarashi K., Fan L., Yang S., Li Y., Jin Y. (2018). Executive function predicts the visuospatial working memory in autism spectrum disorder and attention-deficit/hyperactivity disorder. Autism Res..

[45] Liu T., Liu X., Yi L., Zhu C., Markey P.S., Pelowski M. (2019). Assessing autism at its social and developmental roots: A review of Autism Spectrum Disorder studies using functional near-infrared spectroscopy. NeuroImage.

[46] Ruiz M., Groessing A., Guran A., Koçan A.U., Mikus N., Nater U.M., Kouwer K., Posserud M.B., Salomon-Gimmon M., Todorova B. (2023). Music for autism: A protocol for an international randomized crossover trial on music therapy for children with autism. Front. Psychiatry.

[47] Sokhadze E.M., El-Baz A., Baruth J., Mathai G., Sears L., Casanova M.F. (2009). Effects of low frequency repetitive transcranial magnetic stimulation (rTMS) on gamma frequency oscillations and event-related potentials during processing of illusory figures in autism. J. Autism Dev. Disord..

[48] Ameis S.H., Blumberger D.M., Croarkin P.E., Mabbott D.J., Lai M.C., Desarkar P., Szatmari P., Daskalakis Z.J. (2020). Treatment of Executive Function Deficits in autism spectrum disorder with repetitive transcranial magnetic stimulation: A double-blind, sham-controlled, pilot trial. Brain Stimul..

[49] Ngetich R., Zhou J., Zhang J., Jin Z., Li L. (2020). Assessing the Effects of Continuous Theta Burst Stimulation Over the Dorsolateral Prefrontal Cortex on Human Cognition: A Systematic Review. Front. Integr. Neurosci..

